# Glutamine Synthetase Sensitivity to Oxidative Modification during Nutrient Starvation in *Prochlorococcus marinus* PCC 9511

**DOI:** 10.1371/journal.pone.0135322

**Published:** 2015-08-13

**Authors:** Guadalupe Gómez-Baena, María Agustina Domínguez-Martín, Robert P. Donaldson, José Manuel García-Fernández, Jesús Diez

**Affiliations:** 1 Departamento de Bioquímica y Biología Molecular, Edificio Severo Ochoa, Universidad de Córdoba, Córdoba, Spain; 2 Department of Biological Sciences, The George Washington University, Washington, D.C., United States of America; CEA-Saclay, FRANCE

## Abstract

Glutamine synthetase plays a key role in nitrogen metabolism, thus the fine regulation of this enzyme in *Prochlorococcus*, which is especially important in the oligotrophic oceans where this marine cyanobacterium thrives. In this work, we studied the metal-catalyzed oxidation of glutamine synthetase in cultures of *Prochlorococcus marinus* strain PCC 9511 subjected to nutrient limitation. Nitrogen deprivation caused glutamine synthetase to be more sensitive to metal-catalyzed oxidation (a 36% increase compared to control, non starved samples). Nutrient starvation induced also a clear increase (three-fold in the case of nitrogen) in the concentration of carbonyl derivatives in cell extracts, which was also higher (22%) upon addition of the inhibitor of electron transport, DCMU, to cultures. Our results indicate that nutrient limitations, representative of the natural conditions in the *Prochlorococcus* habitat, affect the response of glutamine synthetase to oxidative inactivating systems. Implications of these results on the regulation of glutamine synthetase by oxidative alteration prior to degradation of the enzyme in *Prochlorococcus* are discussed.

## Introduction

*Prochlorococcus* is a marine cyanobacterium responsible for an important part of the primary production in the intertropical oceans where it is the dominant photosynthetic organism [1–4]. Its ability to cope with the oligotrophic conditions in its natural habitat is a critical part of its life strategy [4–9] and therefore, studying nutrient assimilation is crucial to understand the success of *Prochlorococcus* in the ocean.

Nitrogen is the most important nutrient controlling primary productivity in the marine environment, together with iron and phosphorus [10]. The preferred nitrogen source for all the *Prochlorococcus* strains studied so far is ammonium [11, 12] and the glutamine synthetase/glutamate synthase (GS/GOGAT) pathway is the main route to incorporate it into the carbon backbones. Previous work [13–16] showed that GS from *Prochlorococcus* is not sensitive to some of the classical regulatory pathways found in other cyanobacteria [17]. For instance, GS activity does not respond either to changes in nitrogen or light availability [13, 14] despite the increase in the expression of *glnA* [18], gene encoding for GS subunits, observed under nitrogen limitation. Previous studies from our group demonstrated that GS and isocitrate dehydrogenase (ICDH) are susceptible to metal-catalyzed oxidation (MCO) in vitro [16, 19] as has been described for GS from different organisms [20–27]. Metalloproteins, as GS, are especially sensitive because they possess a metal binding site in their molecular structure to accommodate the metal cofactor (Mg^2+^ or Mn^2+^ in the case of GS) needed in the enzymatic reaction. Cations such as Fe^2+^ and Cu^1+^ can also bind to the metal binding site of the enzyme. These metals are highly reactive with hydrogen peroxide (Fenton reaction) provoking the formation of reactive oxygen species (ROS) that can promote the oxidation of surrounding amino acids in the enzyme (recently reviewed [28]).

Our group described that, in *Prochlorococcus*, this regulatory system consists of two consecutive steps: the enzyme is first inactivated, and then cleaved by endoproteases into several fragments of 40, 32 and <10 kDa [16]. The inactivation is prevented by the presence of the physiological substrates for the GS and the loss of activity can be partially restored by the addition of ATP, reflecting the potential physiological role of this regulatory system in *Prochlorococcus* [16]. Previous results suggested that MCO could be implicated in the regulation of GS under stress conditions such as stationary phase adaptation [16]. The conservation of such a regulatory mechanism in *Prochlorococcus* is a very interesting feature in relation to the marked reduction in the genome size and the consequential streamlining of regulation pathways in this organism [29–34].

The detection of carbonyl groups has been used as a marker for oxidative damage produced under several physiological and pathological conditions [35, 36], since carbonyl derivatives are formed in proteins as a consequence of the MCO reaction. Therefore, in the present work we have first investigated whether MCO promotes the in vitro carbonylation of GS by using two inactivating model systems: NADH/Fe^3+^ and ascorbate/Cu^2+^. Then, we explored the involvement of MCO in the regulation of GS under nutrient starvation. Finally, we further characterized the effect of electron transport inhibitors and darkness on the carbonylation of GS.

## Materials and Methods

### Strains and culturing

*Prochlorococcus marinus* strain PCC 9511 (surface adapted, axenic) was cultured in polycarbonate Nalgene flasks using PCR-S11 medium as described by Rippka and coworkers [37]. Cells were grown at 24°C under continuous blue irradiance (40 μmol quanta m^-2^ s^-1^). Growth was determined by measuring the culture absorbance at 674 nm. Cells at the exponential phase of growth were harvested by centrifugation at 30,100 *g* for 5 min at 4°C. After pouring off most of the supernatant and carefully pipetting out the remaining medium, the pellet was directly resuspended in cold 50 mM Tris-HCl pH 7.5 (2 mL buffer per litre of culture) and stored at –20°C until used.

Experiments requiring darkness, 2,5-dibromo-3-methyl-6-isopropyl-p-benzoquinone (DBMIB) or 3-(3,4-dichlorophenyl)-1,1-dimethylurea (DCMU) were prepared as previously described [13]. For experiments involving nutrient starvation, 10 L cultures at 0.05 units of A_674_ were used. Aliquots of 2 L were centrifuged at 30,000 *g* for 5 min at 24°C, and the pellets obtained were washed and resuspended with PCR-S11 medium lacking one of the nutrients and compared to cells resuspended in complete medium (controls).

### Preparation of crude extracts

Cells were broken by thawing them in the same Tris buffer used for storage. The broken material was centrifuged at 16,100 *g* for 10 min at 4°C and the resulting supernatant was used.

### Inactivation assays

Incubations were prepared as previously described [15]. In the inactivation reactions by the NADH/Fe^3+^ system, crude extracts were incubated at 30°C in a solution containing 0.2 mM FeCl_3_ and 5 mM NADH. In the case of the ascorbate/Cu^2+^ system, incubations were carried out at 4°C in a solution containing 0.2 mM CuCl_2_ and 10 mM ascorbate.

### Enzymatic assays

GS transferase activity was determined as previously described [13].

### Spectrophotometric quantification of carbonyl groups

Carbonyl derivatives were quantified using the method described by Levine [38] with the modifications of Nguyen and Donaldson [39].

### Immunochemical detection of carbonylated proteins

Carbonylated proteins were detected using the Oxyblot Protein Oxidation Detection Kit (Chemicon International). SDS-PAGE was performed as described [40], by loading same amounts of total protein (15 μg) on a MiniProtean III system (Bio-Rad), using 12% resolving and 4% stacking gels. Samples were transferred for 50 min at 150 mA to a nitrocellulose membrane (Sigma) utilizing a Trans-Blot SD system (Bio-Rad). After transfer, the membrane was treated as follows: washing for 15 min with T-TBS buffer (20 mM Tris-HCl pH 7.5 supplemented with 155 mM NaCl and 0.1% Tween 20); blocking with T-TBS containing 5% non fat milk for 1 h and washing twofold for 15 s with T-TBS buffer. Oxidized proteins were detected with anti-1,3 dinitrophenylhydrazone (anti-DNP) antibodies (Chemicon International), diluted 1:150 in 1% non fat milk in T-TBS. Anti-immunoglobulin from rabbit produced in goat, linked with horseradish peroxidase (Bio-Rad) diluted 1:6000 was used as secondary antibody. Supersignal West Pico Chemiluminiscent Substrate (Pierce, Inc.) and Kodak Biomax Light film were used to visualize antibody binding. The membrane was subsequently stripped for 5 min using 3 M guanidine-thiocyanate and reprobed with anti-GS antibodies from *Synechocystis* PCC 6803 (prepared in rabbit and kindly provided by Prof. Florencio and Dr. Muro-Pastor). These antibodies show a high specificity against GS from *Prochlorococcus* cell extracts and have been characterized previously by using rocket immunoelectrophoresis and western blotting in studies with *Prochlorococcus* [13–15, 41]. Molecular masses of proteins were estimated by using molecular mass markers (Bio-Rad, 161–0305, pre-stained, low range).

### Antioxidant activity

The antioxidant activities of extracts obtained from cultures subjected to the different treatments were determined applying the ABTS^+^ radical cation decolorization assay [42]. The stable ABTS^+^ radical cation was generated by incubating 7 mM of ABTS (2,2'-azino-bis(3-ethylbenzothiazoline-6-sulphonic acid)) with 2.45 mM of potassium persulfate for 12–16 h. Then, the mixture was diluted in 50 mM Tris-HCl pH 7.5 until the absorbance at 734 nm reached about 0.7 ± 0.2 units. Equivalent volumes of cells extracts containing 30 μg of protein were added and the absorbance checked after 6 min. The data shown correspond to the percentage of absorbance lost with respect to a control assay with no cell extract added.

### Immunoprecipitation of GS

Cells extracts of *Prochlorococcus* sp. PCC 9511 subjected to different conditions of nutrient starvation and energy deprivation were assessed by GS immunoprecipitation, coupled to the carbonylation assay, SDS-PAGE and western blot analysis. A volume of extract containing 750 μg of protein was mixed with 40 μL of immunoprecipitation buffer (20 mM Tris-HCl, pH 7.5, 1% Triton X-100, 150 mM NaCl, 10% glycerol, 1 mM Na_3_VO_4_, 50 mM NaF, 2 mM EDTA, 1 mM PMSF) with protein G Sepharose (GE Healthcare) and 6 μg of the *Synechocystis* anti-GS antibodies, incubated at 4°C overnight with rotation. Then, the sample was washed with the immunoprecipitation buffer and the pellets resuspended in 50 μL of Tris 50 mM EDTA 1 mM pH 7.4. A western blot using the anti-GS antibodies with aliquots of the same samples (10 μL) was done in order to confirm that GS had been immunoprecipitated.

### Carbonylation of the immunoprecipitated GS

Immunoprecipitated samples were subjected to the carbonylation protocol following the method previously described [43]. 10 μL of these samples were subjected to SDS-PAGE performed on a Mini-Protean III system (Bio-Rad) using 12% resolving and 4% stacking gels. Extracts were transferred to a nitrocellulose membrane (Sigma) utilizing a semidry Trans-Blot Turbo Transfer System (Bio-Rad). Transfer was performed for 30 min at 100 mA. After transfer the membrane was treated as follows: 15 min washing with TBS-T buffer (20 mM Tris-HCl pH 7.4, 150 mM NaCl and 0.1% Tween 20); 2 h blocking with TBS-T containing 1% bovine serum albumin and washing three-fold for 15 min with TBS-T buffer. GS was detected using as primary antibody the anti- *Synechocystis* GSI diluted 1:3000 (v/v) in TBS-T 1% bovine serum albumin (overnight incubation with shaking at 4°C) and washing three-fold for 15 min with TBS-T buffer. Incubation with secondary antibody (goat anti-rabbit-immunoglobulin, linked with peroxidase, Sigma) diluted 1:2000 (v/v) in TBS-T was performed for 30 min with shaking at room temperature followed by three 15 min washing with TBS-T buffer. Then the immunoreacting material was detected by using the ECL Plus Western Blotting Detection System (Amersham), adding 400 μL of solution A supplemented with 400 μL of solution B. The chemiluminescent signal was detected using a LAS-3000 camera (Fujifilm) and images analyzed using Multi-Gauge V3.0 (Fujifilm). Molecular mass was estimated by comparison with molecular markers provided by gTPbio (bioBLU Prestained Protein Ladder).

### Protein concentration determination

Protein concentration of soluble fractions was determined by the Bio-Rad Protein assay, according to the instructions of the manufacturer, using bovine serum albumin as standard. When samples contained SDS, the Micro BCA protein assay (Pierce) was used according to the instructions of the manufacturer, using bovine serum albumin and the appropriate amount of SDS in the standard.

### Statistical analysis

Experiments were carried out at least with three independent biological samples. The results are shown with the standard deviation. Significance of data was assessed by using the Student's T test, and indicated in tables with asterisks: * means *p* value ≤ 0.05; ** means *p* value ≤ 0.01.

## Results

### GS is carbonylated in vitro in the presence of the NADH/Fe^3+^ and the ascorbate/Cu^2+^ MCO systems

**Fig 1:**
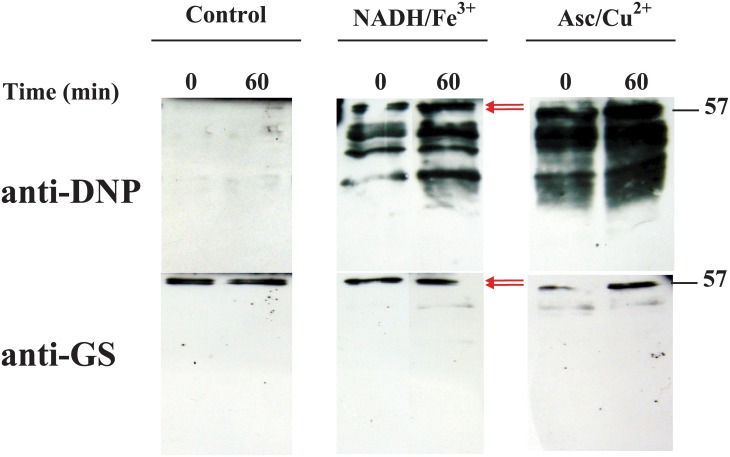
Immunochemical detection of carbonylated proteins. To characterize in vitro the effect of the MCO systems we utilized cell extracts from cultures collected in the logarithmic stage of growth (0.05 units of absorbance at 674 nm). Inactivation assays were prepared as stated in Materials and Methods and samples taken at the indicated times. Oxidized proteins were DNPH derivatized and identified by using anti-DNP antibodies. The same nitrocellulose membrane was stripped and reprobed with anti-GS antibodies. The mass, 57 kDa, indicated on the panels corresponds to the molecular mass of the GS band relative to molecular mass markers. The two arrows indicate the two bands of very similar size obtained as an artifact due to the conditions of the DNPH derivatization.

In order to study whether MCO promotes the carbonylation of the GS from *Prochlorococcus*, cell extracts were incubated in the presence of two inactivating systems that promote the formation of reactive oxygen species: NADH/Fe^3+^ and ascorbate/Cu^2+^; the inactivating effects of both systems have been previously described [15]. Aliquots were taken at 0 and 60 min after addition of the MCO systems and treated as described in Materials and Methods for the immunodetection of oxidized proteins. Carbonylated proteins were detected by western blot, membranes were then stripped and reprobed for GS identification. As expected, the carbonylated proteins increased in the samples exposed to the MCO systems, while in the control samples no bands were detected in our experiments (Fig 1). In Fig 1, zero times correspond to aliquots taken immediately after adding the MCO system (buffer in the case of the controls). The reaction is so rapid that this lapse of time seems to be enough to promote carbonylation in the proteins. This rapid effect was also seen in previous studies when measuring GS inactivation [15, 16]. Once the membranes were re-developed using specific anti-GS antibodies, we observed that this enzyme seems to be one of the proteins carbonylated in the presence of the MCO systems. Using specific anti-GS antibodies, it was also evident a faint band of about 50 kDa in the samples incubated for 60 min in the presence of the MCO systems, likely corresponding to one of the degradation products described in a previous work [16].

It is noteworthy that we detected two bands corresponding to GS, with very similar sizes, when samples were processed to detect oxidized proteins (Fig 1, red arrows). This fragmentation of GS was an artifact induced by the acidic pH of the DNPH dilution buffer which contains TFA, since it also happened when we incubated the extracts with the control buffer without DNPH provided in the Oxyblot kit (not shown).

### Effect of nutrient starvation on the oxidative modification of GS in *Prochlorococcus*

The implication of oxidative modification in the regulation of GS under nitrogen, iron and phosphorus starvation was explored, since these nutrients have been considered the main limiting nutrients in the oligotrophic areas of the oceans [44], and their absence affects the regulation of GS in *Prochlorococcus* [14].

*Prochlorococcus* cultures were subjected to nitrogen, phosphorus and iron starvation as described in Materials and Methods. Samples were collected after 48 h. To determinate the GS sensitivity to MCO inactivation, crude extracts were incubated in the presence/absence of the NADH/Fe^3+^ system. Nitrogen and to a lesser extent iron, starvation had a significant effect, making GS transferase activity more sensitive to MCO (Table 1), while phosphorus starvation did not promote an increase in the inactivation rate of GS.

**Table 1:** Effect of essential nutrient starvation on the inactivation of GS transferase activity by the NADH/Fe^3+^ system.

Culture condition	GS transferase activity (%)	
	No addition	NADH/Fe^3+^
Control	98.16 ± 3.17	44.19 ± 2.21
- N	100.65 ± 0.92	28.34 ± 2.25**
- P	100.00 ± 0.00	44.60 ± 1.66
- Fe	103.50 ± 4.94	38.55 ± 2.17*

Samples were taken after 48 h starvation. Crude extracts were incubated in the presence and in the absence of 5 mM NADH and 0.2 mM FeCl_3_. Results show the transferase activity measured after 60 min incubation. 100% activity corresponds to the activity obtained from each condition at zero time. Data are averages of three independent experiments, determined in triplicate ± standard deviations. T-test significance **p value ≤0.01, *p value ≤0.05.

**Fig 2:**
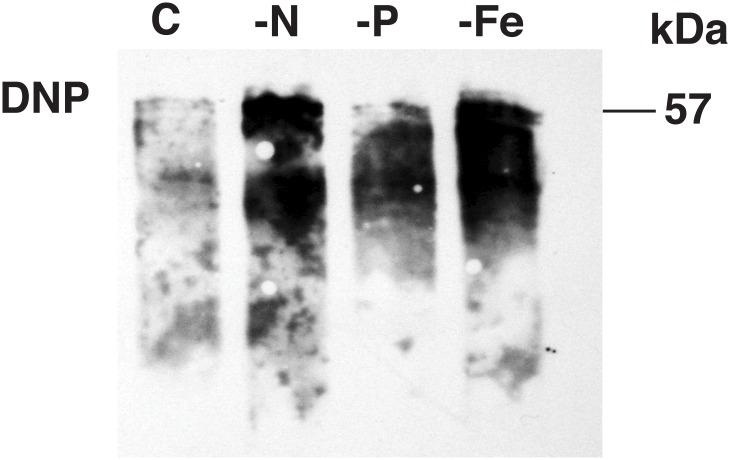
Effect of nutrient starvation on the carbonylation of proteins in *Prochlorococcus* extracts. The cells were subjected to nutrient starvation for 48 h beyond the logarithmic stage of growth. Crude extracts from starved cultures were analyzed for protein oxidation as indicated in Materials and Methods. The same amount of protein (15 μg) was loaded per lane: (C) control, (-N) nitrogen starvation, (-P) phosphorus starvation, (-Fe) iron starvation. Oxidized proteins identified with anti-DNP antibodies. The mass, 57 kDa, indicated on the panel corresponds to the molecular mass of the GS band relative to molecular mass markers.

In order to better understand the effect of nutrient starvation on the cultures, we performed three types of measurement (without MCO treatment): antioxidant activity, concentration of carbonyl derivatives and immunodetection of carbonylated proteins. Antioxidant activity of crude extracts was measured in order to determine whether the inactivation of GS was due to an overproduction of ROS in the cultures subjected to nitrogen starvation. However, these conditions did not significantly alter the antioxidant activity with respect to control cultures. Control samples showed 26.02 ± 2.24% of antioxidant capacity while -N, -P and -Fe showed 21.38 ± 2.64%, 21.29 ± 4.85% and 30.8 ± 2.3% respectively, using 30 μg of protein in the assay. The concentrations of carbonyl derivatives were also quantified (Table 2). Nitrogen starvation promoted the formation of an amount of carbonyl derivatives 3-fold higher than that measured in the control cultures (Table 2). Finally, immunochemical detection of oxidized proteins was carried out in crude extracts obtained from cultures subjected to nutrient starvation (Fig 2), loading the same amount of protein per lane (15 μg). There was a clear increase of carbonylated proteins under nitrogen and iron limitation, indicating the relationship between nutrient starvation and the carbonylation state of proteins. Slight carbonylation was also detected in the case of phosphorus starvation.

**Table 2:** Effect of essential nutrient starvation on the carbonyl derivatives concentrations.

Culture condition	DNP concentration (%)
Control	100 ± 32.8
- N	316 ± 18.0 **
- P	150 ± 26.3
- Fe	159 ± 15.1 *

Starved cultures were prepared as indicated in Materials and Methods. Samples were taken after 48 h of treatments in the indicated conditions. Crude extracts were used to quantify carbonyl derivatives concentrations as described in Materials and Methods. Measurements were normalized to protein concentration in the corresponding cell extract. Data are averages of three independent experiments, determined in triplicate ± standard deviations. 100% corresponds to the average concentration obtained from a control culture incubated in the same conditions as the starved cultures for 48 h (4.30 nmol DNP/mg protein). T-test significance **p value ≤0.01,* p value ≤0.05.

### Relationship between the redox balance and the carbonylation of GS

**Fig 3:**
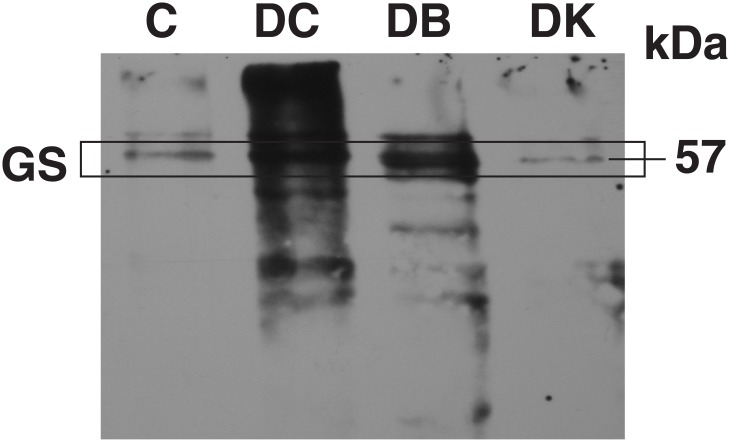
Effect of DCMU, DBMIB and darkness on the carbonylation of proteins in *Prochlorococcus* extracts. The cells were incubated with the inhibitors and under darkness for 24 h beyond the logarithmic stage of growth. DNPH derivatized proteins detected as described in Materials and Methods. The same amount of protein (15 μg) was loaded per lane: (C) control, (DC) DCMU, (DB) DBMIB, (DK) darkness. Oxidized proteins were identified by using anti-DNP antibodies. The mass, 57 kDa, indicated on the panel corresponds to the molecular mass of GS band relative to molecular mass markers.

Previous results indicate that DCMU, an inhibitor of the photosynthetic electron transport chain, can trigger the oxidative modification of GS in *Prochlorococcus* [16], therefore, we quantified the carbonyl derivative concentrations in cultures treated with DCMU and DBMIB, two inhibitors of the photosynthetic electron chain, acting respectively before and after the plastoquinone (PQ) pool [45]. These results were also compared with those obtained when cells were cultured under darkness (Table 3). Carbonyl concentrations increased in extracts from DCMU treated cultures, while in those treated with DBMIB and those subjected to darkness, carbonyl concentration did not change with respect to the control cultures. We also analyzed protein carbonylation under these conditions. Extracts were treated for carbonyl detection as described in Materials and Methods and western blots were carried out. In the case of control and dark cultures, the protein oxidation rate was almost identical (Fig 3). However, DCMU and DBMIB treatments increased the oxidation of proteins relative to control cultures, more so in the case of DCMU.

**Table 3:** Effect of the redox state of the cells on the carbonyl derivatives concentrations.

Culture condition	DNP concentration (%)
Control	100 ± 6.25
DCMU 0.3 μM	122 ± 5.16**
DBMIB 0.06 μM	99 ± 9.75
Darkness	92 ± 3.17

Samples were taken after 24 h treatments in the indicated conditions. Crude extracts were used to quantify carbonyl derivatives concentrations as described in Materials and Methods. Measurements were normalized to protein concentration in the corresponding cell extract. Data are averages of three independent experiments, determined in triplicate ± standard deviations. 100% corresponds to the average concentration obtained from a control culture incubated for 24 h in the same conditions as the treated cultures (24.01 nmol DNP/mg protein). T-test significance **p value ≤0.01.

### Immunoprecipitation of GS from Prochlorococcus PCC 9511 under different conditions

In order to confirm that GS is one of the enzymes carbonylated in our study, we performed the immunoprecipitation and subsequent carbonylation analysis of this enzyme from extracts of *Prochlorococcus* PCC 9511 subjected to different conditions. As quality control step, an aliquot of the immunoprecipitation mixture was taken and a western blot using specific anti-GS antibodies was performed before carbonylation analysis (not shown). This western revealed a single band with a molecular weight of ca. 57 kDa. This result demonstrated that the protein obtained through the immunoprecipitation was GS.

**Fig 4:**
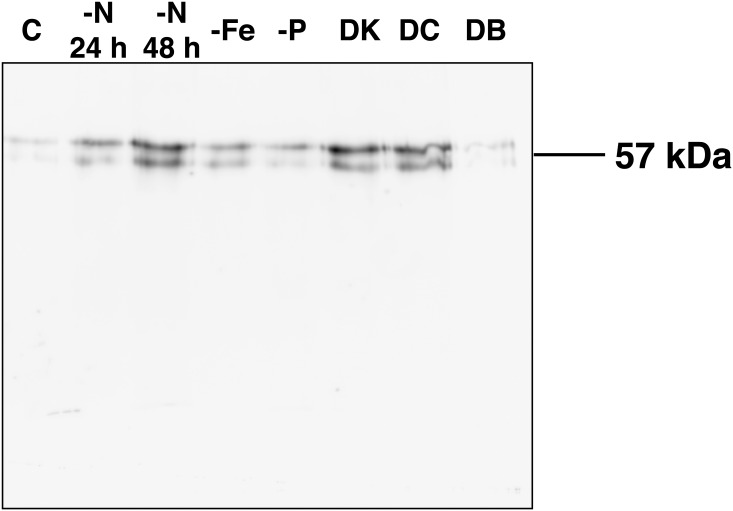
Immunoprecipitation of GS under different experimental conditions. The cells were incubated under the different conditions for 24–48 h beyond the logarithmic stage of growth. After this period, GS was immunoprecipitated and analyzed for protein oxidation detection as described in Materials and Methods. Oxidized GS was identified by using anti-DNP antibodies: (C) control, (-N 24 h) and (-N 48 h) nitrogen starvation at 24 and 48 h, (-Fe) iron starvation, (-P) phosphorus starvation, (DK) darkness, (DC) DCMU, (DB) DBMIB.

Then, the immunoprecipitated GSs from different conditions were subjected to immunochemical detection of carbonylation (Fig 4). When anti-DNP antibodies were used, a double band with the molecular weight of ca. 57 kDa was detected, showing that GS is carbonylated under all three nutrient starvation conditions examined in the present work, while a faint band was detected in the control sample. The carbonylation level of GS increased with the time of N starvation (Fig 4, lanes 2 and 3); this suggests that GS carbonylation is a process actually involved in the physiological regulation of this enzyme in *Prochlorococcus*. Carbonylation was also evident in the case of DCMU addition, confirming our previous findings, and darkness, while similar levels to control were obtained in the case of DBMIB. These results support the hypothesis that GS from *Prochlorococcus* PCC9511 is carbonylated under the different treatments studied in the present work.

## Discussion

During the last 30 years, research on protein modification prior to degradation has evolved significantly. Initial investigations in *Escherichia coli* and *Klebsiella aerogenes* showed that GS was one of the proteins rapidly degraded when cells were subjected to nitrogen starvation, this process being under metabolic control [25, 46]. These works established the hypothesis that oxidative modification of specific proteins could function as a mechanism for cellular regulation (reviewed in [47]), and not only as an aberrant consequence of the metabolism which organisms have to cope with, as initially expected.

Previous studies from our group have demonstrated that GS from *Prochlorococcus* is sensitive to MCO inactivation and subsequent degradation, showing that changes in the redox state of the cell and the aging of the cultures could act as triggering signals for the MCO modification of this key enzyme [15, 16]. In the present work we further characterized the effect of changes in the cell redox state, also testing whether limitation of essential nutrients could initiate oxidative modification of GS in *Prochlorococcus*.

Adaptation to oligotrophic conditions seems to be one of the main selective forces in *Prochlorococcus* strains [4, 5, 11, 48, 49]. Cyanobacteria have evolved different mechanisms to cope with nutrient starvation. One of the most important ways to conserve energy is to recycle amino acids obtained from the degradation of nonessential proteins [50], which could act as substrates for the synthesis of proteins needed for adaptation to nutrient limitation [51]. MCO systems leading to inactivation and later degradation of proteins might be particularly useful in organisms such as *Prochlorococcus*, for which recycling of organic matter is most important. This hypothesis fits nicely with previous reports showing the importance of amino acid [52] and nucleotide [53] recycling in *Prochlorococcus*.

Our results suggest that GS from *Prochlorococcus* could be oxidatively regulated under nitrogen starvation. This is particularly interesting, given that the GS inactivating factors described in other cyanobacterial strains (IF7 and IF17) seem to be absent in the *Prochlorococcus* genus [54]. In a recent work we showed that GS is one of the proteins affected by redox modification under nitrogen starvation [41]. In the present work we show that the concentration of carbonyl derivatives in nitrogen-starved cultures is three-fold higher than that in the control cultures, reinforcing the hypothesis of nitrogen starvation promoting oxidative modification of proteins. Furthermore, GS in nitrogen-starved cultures is more sensitive to MCO inactivation (Table 1) and is carbonylated (Fig 4). Previous results have shown that GS activity and protein does not change significantly under nitrogen starvation [13] while *glnA* expression is upregulated under nitrogen starvation [18]. Since the concentration of a given protein is the result of synthesis and degradation, regulation by oxidative modification fits nicely within this picture: GS would be irreversibly tagged for degradation by carbonylation, hence synthesis would need to be increased to keep the enzyme at the same concentration in the cell. Further studies on protein turnover kinetics would offer more information about this issue.

Iron starvation also seems to be a signal promoting the oxidative modification of GS (Fig 4). This is not unexpected based on the results from studies focused on the relationship between iron and redox homeostasis in cyanobacteria [55–57]. In *Prochlorococcus*, GS activity decreases drastically in iron-starved cultures, along with a marked decrease in total protein concentration [14]. The previous studies along with the observations presented here indicate that GS could be oxidatively modified and degraded under iron starvation, since GS activity is more sensitive to MCO inactivation and the GS protein is carbonylated under these conditions.

We also explore if oxidative modification could be involved in the regulation of GS under phosphorus starvation since previous work demonstrated that GS activity decreases under P limitation [13]. Fig 2 shows considerable carbonylation correlating well with the carbonyl derivatives quantification in the extract (Table 2). However, the activity is not affected by the addition of MCO (Table 1) and the level of carbonylation in the immunoprecipitated GS is similar to that in the control samples. Hence, the lost in GS activity observed under phosphorus starvation [13] can not be clearly attributed to oxidative modification.

The oxidative modification of molecules induced by ROS has been related to a wide range of physiological processes. Photosynthetic organisms produce ROS continuously through the leakage of electrons from the electron transport chain, particularly when the energy absorbed exceeds the capacity of electron transfer. The inhibition of the electron transport exerted by DCMU and DBMIB promotes an increase on the production of ROS [58]. In a previous study, we showed that the presence of DCMU, DBMIB and darkness made GS transferase activity more sensitive to the MCO inactivating systems, and degradation of GS was enhanced in the presence of DCMU. Antioxidant capacity was also altered in the presence of DCMU, being 50% lower than that observed in the control samples [16]. In the present work we observed an increase in the concentration of carbonyl derivatives (Table 3) and we demonstrated that GS is carbonylated in DCMU-treated cultures (Figs 3 and 4). GS was also carbonylated under darkness and at a lower extent in the presence of DBMIB, suggesting its modification by ROS (Figs 3 and 4). However, the different behaviour of the two inhibitors was striking. Recent studies have proposed the existence of a plastoquinol terminal oxidase in some *Prochlorococcus* strains, including the strain used in this work [59]. One of the functions reported for this protein is the modulation of the PQ pool redox state: the plastoquinol terminal oxidase catalyzes the electron transfer to oxygen [60] when the PQ pool is highly reduced, as happens in the presence of DBMIB. The existence of this activity could explain the lower damaging effect promoted by DBMIB compared with that of DCMU. GS carbonylation under darkness (Fig 4) is quite unexpected. More experiments focused on the proteases responsible for degradation are needed to confirm if is a consequence of the accumulation of carbonylated GS due to restricted degradation under energy deprivation. These results together with those obtained in a previous work [16], support the hypothesis that changes in the redox state of the cells promote the oxidation of GS in *Prochlorococcus*.

It is widely accepted that many different stimuli activate the production of ROS as part of the intracellular responses to changes in environmental conditions. Redox regulatory mechanisms present a clear advantage: they are very rapidly triggered, since ROS themselves act as messenger molecules. In *Prochlorococcus*, redox regulated degradation of proteins could help to cope with starvation for essential nutrients, such as nitrogen and iron, through amino acid recycling thus contributing to a better adaptation to the natural limitations of the *Prochlorococcus* habitat.

The present work is a step forward in the characterization of the regulation of GS by oxidative modification. In previous works we have characterized the inactivation [15] and degradation of GS [16], using in vitro models and different culture conditions. In the present work, we intended to investigate whether GS is carbonylated during oxidative modification since carbonylation has been described as a tagging system prior degradation. Hence, in the first section we show that MCO in vitro systems promote extensive carbonylation. Then, we explore GS carbonylation under different culture conditions (without adding MCO systems) to show that GS carbonylation occurs in some cases, being particularly clear the case of nitrogen starvation, iron starvation and DCMU.
